# The complete chloroplast genome of *Atractylodes macrocephala* Koidz. (Asteraceae)

**DOI:** 10.1080/23802359.2020.1763867

**Published:** 2020-05-13

**Authors:** Honghui Li, Guo Yang

**Affiliations:** School of Life Science, Shaoxing University, Shaoxing, China

**Keywords:** *Atractylodes macrocephala*, complete chloroplast genome, phylogenetic analysis

## Abstract

*Atractylodes macrocephala* Koidz. is a traditional medicinal plant, distributing in China, Korea, and Japan. Here, we assembled and characterized the complete chloroplast (cp) genome of *A. macrocephala* using Illumina sequencing data. The plastome is 153,256 bp in length, consisting of a pair of inverted repeats (IRs) of 25,146 bp, separated by a large single-copy (LSC) region of 84,290 bp and small single-copy (SSC) region of 18,674 bp. A total of 125 genes were identified from the genome, including 88 protein-coding genes, 29 tRNA genes, and eight rRNA genes. The overall GC content of the genome is 37.7%. The phylogenetic analysis based on 42 complete cp sequences revealed that *A. macrocephala* is closely related to *A. chinensis* and *A. lancea*.

*Atractylodes* DC. is an important genus of Asteraceae. The genus *Atractylodes* contains seven species, distributing in China, Korea, and Japan. Several *Atractylodes* species have been widely used in traditional medicine. *Atractylodes macrocephala* Koidz., the best known plants in this genus, has a long history being used as medicine. The dried rhizomes of *A. macrocephala* are called ‘Baizhu’ in Chinese (Mizukami et al. 1998). However, there is little research about the complete chloroplast (cp) genome of *A. macrocephala.* In this study, the complete cp genome of *A. macrocephala* were sequenced. Meanwhile, we characterized the plastome based on the genome skimming sequencing data.

Fresh leaves of *A. macrocephala* were collected in Xinchang Baizhu Planting Base (Zhejiang, China; 120°E, 29°N). Plant specimens were conserved in the Herbarium of Shaoxing University (accession number: SXU-20190415AM02). The total genomic DNA was extracted using the Plant DNA Mini Kit (Genepioneer, Nanjing, China). The whole genome sequencing was conducted by Nanjing Genepioneer Biotechnologies Inc. on the Illumina Hiseq platform. The filtered reads were assembled using the program SPAdes assembler 3.10.0 (Bankevich et al. 2012). Annotation was performed using the DOGMA (Wyman et al. 2004) and BLAST search.

The complete cp genome of *A. macrocephala* (GenBank accession number: MN117071) was 153,256 bp in length, with a GC content of 37.7%. The complete cp genome shows a typical quadripartite structure with a pair of inverted repeats (IRs) of 25,146 bp, separated by a large single-copy region (LSC) of 84,290 bp and a small single-copy region (SSC) of 18,674 bp. A total of 125 genes were identified from *A. macrocephala* cp genome, including 88 protein-coding genes, 29 tRNA genes, and eight rRNA genes. Six protein-coding genes, five tRNA genes, and four rRNA genes are duplicated in the IR regions. Fourteen genes contained one intron, and two genes (clpP and ycf3) contained two introns. The corresponding values in IR, LSC, and SSC regions are 43.18%, 35.8%, and 31.57%, respectively.

The phylogenetic analysis was based on the cp genome sequence of *A. macrocephala*, together with 40 species of Asteraceae downloaded from GenBank. *Daucus carota* and *Panax ginseng* were selected as the outgroup. MAFFT v7.307 (Katoh and Standley 2013) was used to align the sequence. The maximum-likelihood method was used to infer the phylogenetic relationship, and the phylogenetic tree was constructed using FastTree version 2.1.10 (Price et al. 2010). The phylogenetic analysis shows that all representatives of *Atractylodes* are clustered into one monophyletic clade with a high bootstrap support value. The results suggest *A. macrocephala* is closely related to *A. chinensis* and *A. lancea* (Figure 1). The complete cp genome sequence will provide a useful resource for conservation genetics of *A. macrocephala*, and the phylogenetic studies of Asteraceae.

**Figure 1.**
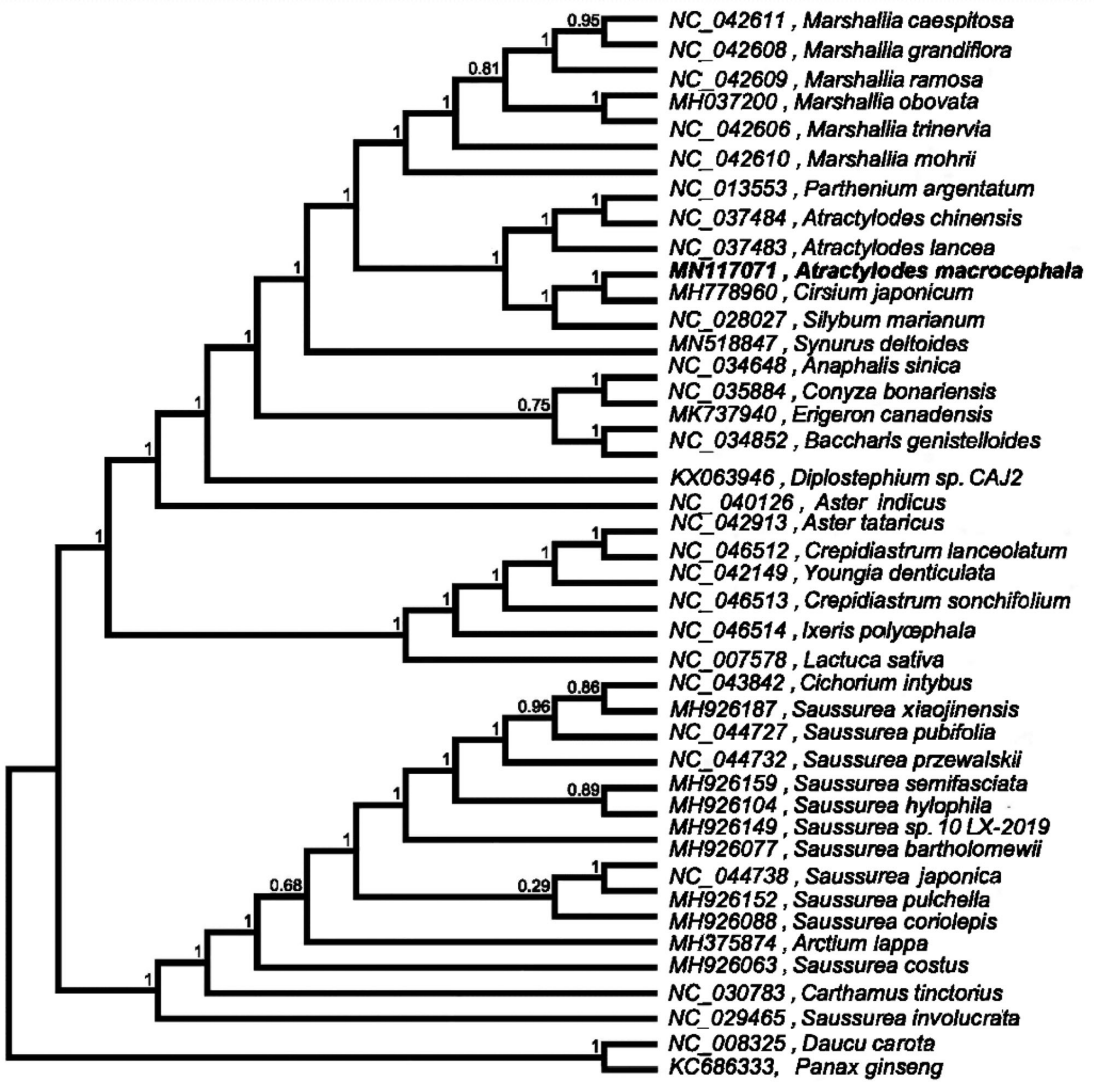
Phylogenetic tree inferred using the maximum-likelihood method based on the complete chloroplast genome of 40 Asteraceae species. *Daucus carota* and *Panax ginseng* were selected as the outgroup. A total of 1000 bootstrap replicates were computed, and the bootstrap support values are shown at the branches. Genbank accession numbers were shown.

## Data Availability

The data that support the findings of this study are openly available in [NCBI] at [https://www.ncbi.nlm.nih.gov/search/all/?term=MN117071], reference number [MN117071].

## References

[CIT0001] Bankevich A, Nurk S, Antipov D, Gurevich AA, Dvorkin M, Kulikov AS, Lesin VM, Nikolenko SI, Pham S, Prjibelski AD, et al. 2012. SPAdes: a new genome assembly algorithm and its applications to single-cell sequencing. J Comput Biol. 19(5):455–477. 22506599 10.1089/cmb.2012.0021 PMC3342519

[CIT0002] Katoh K, Standley DM. 2013. MAFFT multiple sequence alignment software version 7: improvements in performance and usability. Mol Biol Evol. 30(4):772–780. 23329690 10.1093/molbev/mst010 PMC3603318

[CIT0003] Mizukami H, Shimizu R, Kohjyouma M, Kohda H, Kawanishi F, Hiraoka N. 1998. Phylogenetic analysis of *Atractylodes* plants based on chloroplast trnK sequence. Biol Pharm Bull. 21(5):474–478. 9635503 10.1248/bpb.21.474

[CIT0004] Price MN, Dehal PS, Arkin AP. 2010. FastTree 2–approximately maximum-likelihood trees for large alignments. PLOS One. 5(3):e9490. 20224823 10.1371/journal.pone.0009490 PMC2835736

[CIT0005] Wyman SK, Jansen RK, Boore JL. 2004. Automatic annotation of organellar genomes with DOGMA. Bioinformatics. 20(17):3252–3255. 15180927 10.1093/bioinformatics/bth352

